# Recurrence of complete heart block in pregnancy

**DOI:** 10.1016/j.hrcr.2021.07.002

**Published:** 2021-07-22

**Authors:** Daniel Young, Naga Sai Shravan Turaga, F.N.U. Amisha, Kevin Hayes, Hakan Paydak, Subodh R. Devabhaktuni

**Affiliations:** ∗ University of Arkansas for Medical Sciences, Little Rock, Arkansas; † Texarkana Cardiology Associates, Texarkana, Texas

**Keywords:** Complete heart block, Pacemaker, Pregnancy, Recurrent complete heart block, Transient complete heart block

## Introduction

The incidence of complete heart block (CHB) has been reported to be 1 in 15,000–20,000 live births. According to a study done by Reid and colleagues,^1^ 30% of cases of congenital heart block remain undiscovered until adulthood. Hence, most CHBs found in pregnancy are usually congenital heart blocks that are diagnosed for the first time during the first pregnancy or puerperium. However, acquired CHB usually occurs after 50 years of age.^2^ Management of maternal CHB requires a multidisciplinary approach involving cardiologists, obstetricians, anesthesiologists, and neonatologists.

## Case report

**Figure 1:**
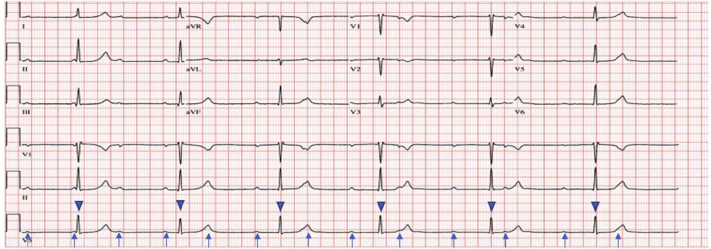
Electrocardiogram on admission demonstrating complete heart block (*arrows* indicate P waves; *arrow heads* indicate QRS complex).

A 37-year-old woman at 30 weeks’ gestational age presented to an outside hospital with exertional dyspnea, fatigue, and intermittent palpitations for 1 week. An electrocardiogram (ECG) was done, which showed a CHB, and the patient was transferred to our facility for further management. She had no chest pain, orthopnea, paroxysmal nocturnal dyspnea, dizziness, syncope or presyncope episodes, rash, joint pains, recent sick contacts, outdoor travel, or drug use. Physical examination was significant for bradycardia (heart rate 40s), stable blood pressure, regular heart sounds, normal jugular venous pulse, and gravid uterus consistent with the gestational age. Fetal heart tones were reassuring, without fetal bradycardia or decelerations. Laboratory data showed mild leukocytosis (15,000/mm^3^), troponin I <0.03 ng/mL (reference normal <0.04), thyroid stimulating hormone mildly elevated to 5.9 (reference normal 0.34–5.60), and T4 of 0.77 (reference normal 0.58–1.64. ECG and telemetry showed CHB (Figure 1). Transthoracic echocardiogram showed no evidence of structural heart disease or valvular abnormalities with normal left ventricular systolic function.

As we have reported in a previous case report,^3^ she had a similar presentation during her previous pregnancy at 26 weeks, with increasing exertional dyspnea and lightheadedness without syncope. The ECG at that time showed CHB, and her transthoracic echocardiogram was unremarkable. Laboratory investigations showed mild leukocytosis (12,000/mm^3^), troponin I 0.24, C-reactive protein 6.2 mg/L, erythrocyte sedimentation rate 19 mm/h, thyroid stimulating hormone 2.36, antinuclear antibodies <1:80, negative Lyme serology. She underwent a treadmill stress test (as per modified Bruce protocol), during which her heart rate increased to 109 beats per minute, and the conduction improved to 2:1 atrioventricular (AV) block. Etiology during that pregnancy was thought to be idiopathic, but there was a possibility of viral myocarditis secondary to an exposure to a toddler experiencing a viral exanthem 10 days before her presentation (hand, foot, and mouth disease). The pregnancy and postpartum course after that was uncomplicated, and the patient reported that she has not had similar episodes until the current presentation. She was followed up periodically in the clinic with ECGs and Holter monitoring and there was no evidence of CHB in between her pregnancies (Supplemental Figure).

**Figure 2:**
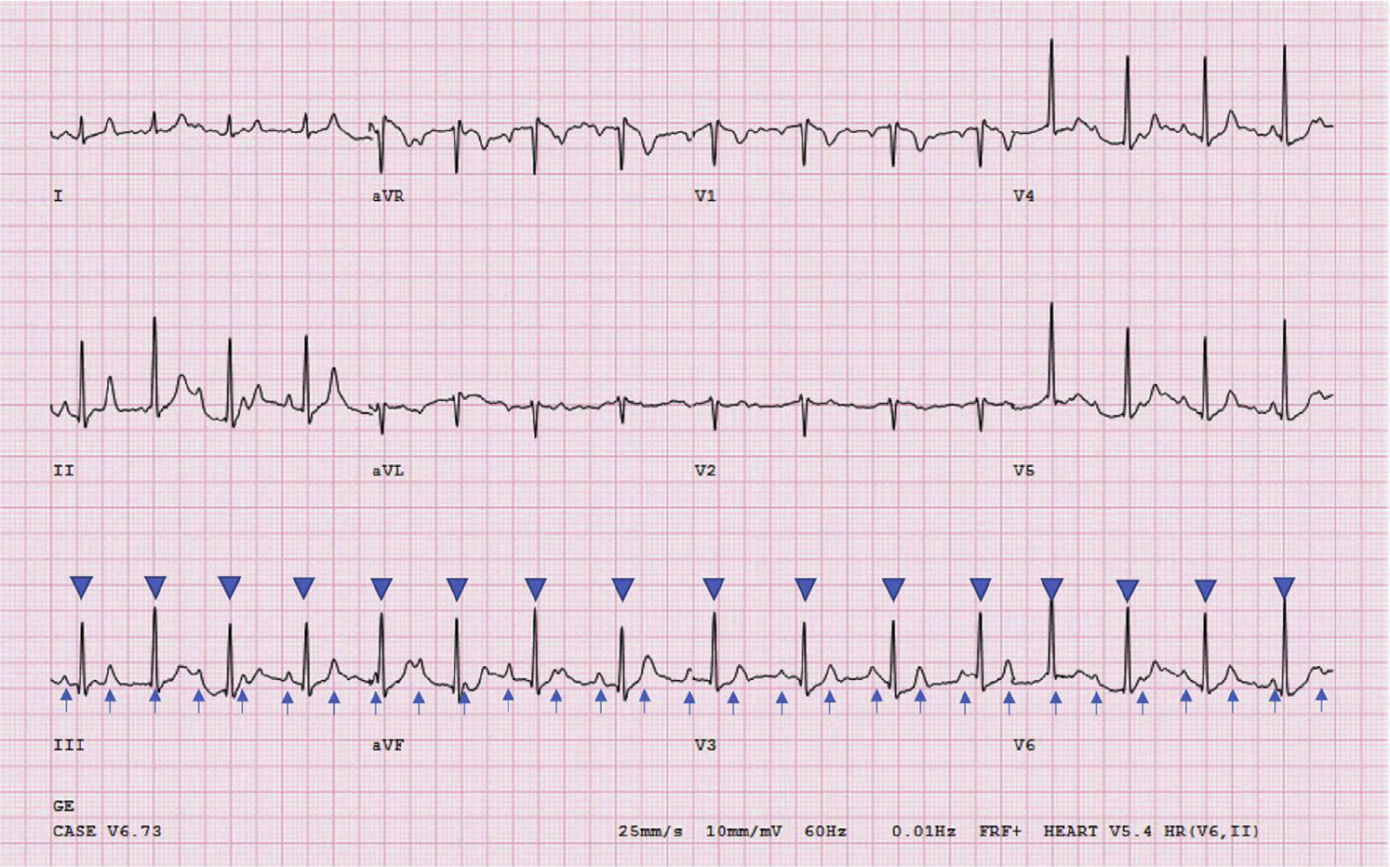
Stress electrocardiogram during the admission in her second pregnancy demonstrating irregular R-R intervals suggestive of intermittently conducting beats through the atrioventricular node (*arrows* indicate P waves; *arrow heads* indicate QRS complex).

With a history of the CHB in a previous pregnancy and now a recurrence, a risk assessment of CHB was made by exercise treadmill testing (as per Naughton protocol). During exercise, she maintained normal blood pressure and was asymptomatic. AV conduction also improved. This was exemplified by irregular R-R intervals, signifying that the patient was no longer in CHB, with some beats conducting through the AV node (Figure 2). The test was stopped once chronotropic competence was achieved. She also received 2 doses of empiric betamethasone for fetal lung maturity in case atropine might be required, which could precipitate premature labor.

**Figure 3:**
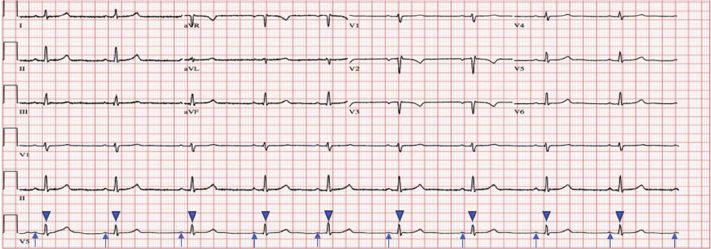
Electrocardiogram obtained postpartum demonstrating sinus rhythm replacing the previous complete heart block (*arrows* indicate P waves; *arrow heads* indicate QRS complex).

The patient remained in the hospital for a total of 3 days with continuous telemetry monitoring, which continued to show CHB with heart rates as low as 30–40 beats per minute while sleeping; however, she remained asymptomatic. The patient had an ECG at an outside facility 2 weeks after discharge, which showed normal sinus rhythm. Since the patient lived a significant distance from our institution, she was followed up via telemedicine 2 months postdischarge. She stated that she had remained asymptomatic. The remainder of her pregnancy progressed without complications. She underwent vacuum-assisted vaginal delivery without neuraxial anesthesia at 39 weeks’ gestation and had no episodes of maternal CHB during labor or the peri- or postpartum periods (Figure 3). The newborn did not show any AV conduction abnormalities following delivery. The patient also received tubal ligation after delivery in order to prevent future episodes of CHB in pregnancy.

## Discussion

Our patient had 1 uneventful pregnancy followed by 2 consecutive pregnancies with CHB, making it most likely an acquired CHB of pregnancy. Although new-onset CHB is rare in pregnancy, there have been a few proposed associations, including metabolic disturbances, autoimmune diseases like systemic lupus erythematosus or sarcoidosis, ischemic heart disease, congenital heart disease, prior cardiac surgery, valvular heart disease, cardiomyopathy, or infections like Lyme disease.^4^, ^5^, ^6^ Most of the workup was unremarkable in our patient with structurally and functionally normal heart, leading to the belief that this CHB was most likely idiopathic. The pathogenesis of an acquired CHB in pregnancy is not fully understood. However, some experts have hypothesized it may be secondary to atrial stretch caused by an increase in blood volume as the pregnancy progresses.^7^ During pregnancy, there is an increase in plasma volume by 50% and red blood cell volume by 30%.^7^ In accordance with Frank-Starling law, the increased volume could cause enough atrial stretch in some patients to lead to a delay in conduction, resulting in CHB. Another proposed mechanism is estrogen-mediated effects on intracellular signaling in cardiac myocytes. GPR30 is a newly discovered estrogen receptor that has structural and functional overlap with beta adrenergic receptors via protein kinase A and G protein–coupled phosphorylation.^8^ There have been marked phenotypic variations in response and sensitivity to estrogen by various tissues and among various species, including humans, which are most likely determined by genetics. This could serve as another possible explanation for the rare occurrence of CHB in pregnancy. This is further supported by the fact that estrogen levels steadily rise in pregnancy, peaking in the third trimester, and that CHB occurred in the late second and third trimester in our patient.

In the setting of CHB during pregnancy, it is important to first stratify patients as symptomatic or asymptomatic. Pacing methods (permanent or temporary) may be indicated for patients that are symptomatic from their CHB (syncope, palpitations, increased shortness of breath, etc). It has been shown in a comprehensive analysis of pregnant patients with CHB by Hidaka and colleagues^9^ that most asymptomatic cases do not require pacing during labor and delivery. In the setting of a patient with CHB that is symptomatic, however, a temporary pacemaker may be required to at least offload the cardiac demand associated with the labor process. Permanent pacing may be considered in the postpartum setting for patients who continue to have a CHB or have symptoms secondary to bradycardia.^10^ The next step for these patients is to assess the prognostic implications of the block. This can be assessed with atropine at the bedside or exercise stress test to evaluate for appropriate elevation in heart rate after the administration of the medicine or beginning exercise. If either of these tests produce an improvement in heart rate, the block is likely at the AV node (intranodal). These patients are often asymptomatic and better able to respond appropriately to increased demand or stress. Their ECG will typically show a narrow QRS pattern as well, representing a junctional escape rhythm. However, if there is no improvement via these methods, the block is likely infranodal and will require pacing assistance owing to patients’ being symptomatic from an inability to increase their heart rate in response to demand. In contrast to intranodal CHB, these infranodal patients often have a wide QRS on ECG, representing a ventricular escape rhythm.^10^^,^^11^

We decided to have our patient walk on a treadmill, as she was confident that she could do so and had remained asymptomatic during her hospital stay. We also decided to not use atropine in the setting of pregnancy owing to an avoidable impact on the fetus, who had remained unaffected by the maternal heart block. During our patient’s exercise, her AV conduction improved, which was proven by irregular R-R intervals on ECG. This ECG does not support CHB and likely represents improvement to high-grade AV block with some P waves that are able to conduct through the AV node. These findings are consistent with transient CHB.

As CHB patients progress to delivery, the preferred mode in the available studies of these patients is vaginal delivery.^12^ Patients in labor with CHB should not receive neuraxial anesthesia if at all possible owing to the unavoidable sympathetic blockade. Efforts should be made with CHB patients to avoid medications and procedures that could result in bradycardia owing to the risks of complications like severe hypotension or asystole that endanger the lives of both mother and fetus.^12^ Published literature on CHB in pregnancy recommend alternatives like incremental epidural or low-dose combined spinal and epidural anesthesia during vaginal delivery.^13^ Patients with CHB who require cesarean delivery have been shown to tolerate general anesthesia with or without epidural or combined spinal-epidural anesthesia techniques.^13^ The chosen anesthetic technique in this situation should be the one with the least expected impact on hemodynamics.

In this case report, the patient delivered a healthy infant without neuraxial block. The newborn had normal Apgar scores and did not show signs of any conduction abnormalities.

The recurrent nature of our patient’s CHB only during pregnancy is not well documented in the literature. This phenomenon in our patient further supports the hypothesis that hemodynamic/hormonal changes during pregnancy may be risk factors for the development of a CHB in some patients. More studies are required for further characterization, including risk stratification and cytogenetics to elaborate the proposed mechanism.

## Conclusion

New-onset acquired transient CHB in pregnancy is a poorly understood and rare phenomenon. We believe this is the first reported case of recurrent transient CHB during pregnancy. Our group also reported the case of transient CHB in this same patient in 2017.^3^ At that time, the CHB was hypothesized to be secondary to possible myocarditis from a viral exanthem. With the recurrence of the CHB in her next pregnancy, we now believe it is a pregnancy-related transient event, which we hypothesize is secondary to conduction defects from physiological atrial stretching during pregnancy or estrogen-mediated cellular signaling changes. In addition, following both pregnancies the patient had 1:1 AV conduction without any indication of even a first-degree AV block. Further research is needed to establish a mechanism of acquired CHB in pregnancy. CHB is usually well tolerated in pregnancy and rarely requires pacing. Sympathetic blockade secondary to neuraxial blockade during delivery can cause bradycardia, resulting in severe hypotension or asystole and threatening the safety of the mother and fetus. Alternatives like combined spinal and epidural anesthesia should be considered for vaginal deliveries. CHB patients requiring cesarean deliveries have been shown to tolerate general anesthesia with or without epidural or combined spinal and epidural anesthesia. Our patient tolerated CHB well and had an uncomplicated course, with the successful vaginal delivery of a healthy infant without any conduction abnormalities.
