# Supplementary material for: Recurrence of complete heart block in pregnancy
Source: HeartRhythm Case Rep. 2021 Jul 22;7(10):679–82. doi: 10.1016/j.hrcr.2021.07.002 (PMC8530940; doi:10.1016/j.hrcr.2021.07.002)

Supplemental figure: Holter monitor done few months after her first delivery showed no evidence of AV heart block at various heart rates.


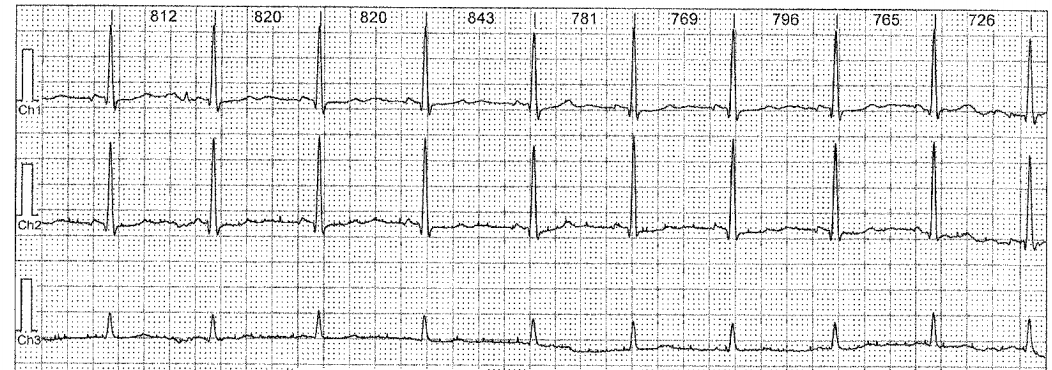


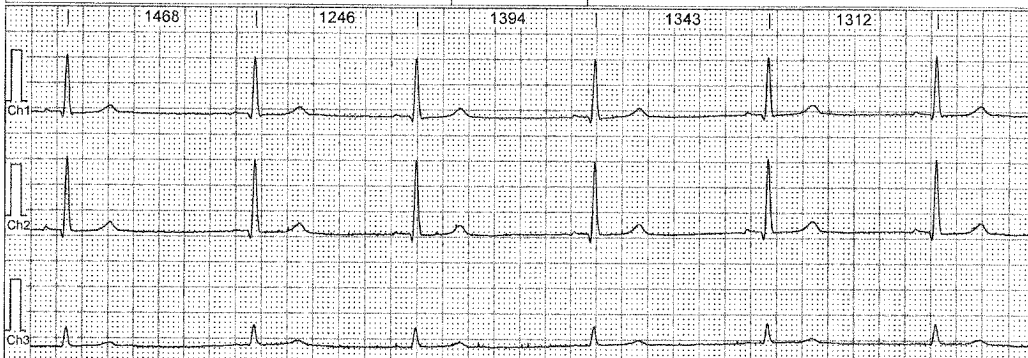

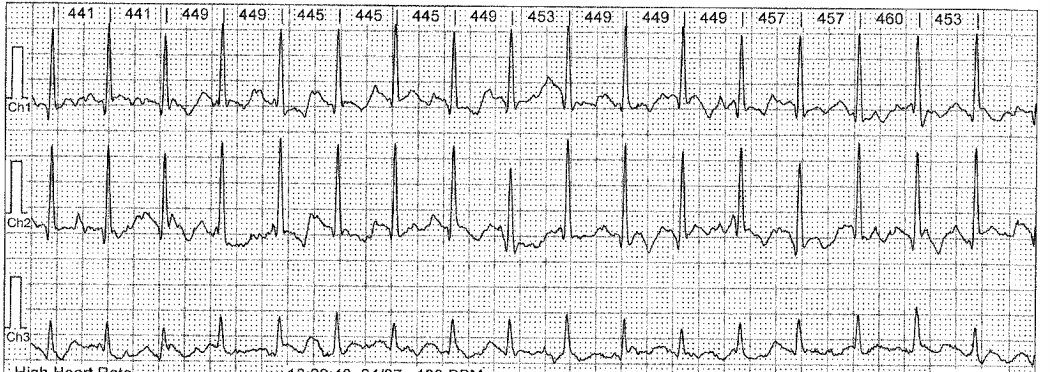

Supplement: Supplemental figure [file mmc1.docx]
